# The G32E Functional Variant Reduces Activity of PPARD by Nuclear Export and Post-Translational Modification in Pigs

**DOI:** 10.1371/journal.pone.0075925

**Published:** 2013-09-18

**Authors:** Yanyu Duan, Bertram Brenig, Xiaohui Wu, Jun Ren, Lusheng Huang

**Affiliations:** 1 Key Laboratory for Animal Biotechnology of Jiangxi Province and the Ministry of Agriculture of China, Jiangxi Agricultural University, Nanchang, China; 2 Institute of Veterinary Medicine, Georg-August-University of Göttingen, Göttingen, Germany; 3 Institute of Developmental Biology & Molecular Medicine, Fudan University, Shanghai, China; Wageningen UR Livestock Research, The Netherlands

## Abstract

Peroxisome proliferator-activated receptor beta/delta (PPARD) is a crucial and multifaceted determinant of diverse biological functions including lipid metabolism, embryonic development, inflammatory response, wound healing and cancer. Recently, we proposed a novel function of porcine PPARD (sPPARD) in external ear development. A missense mutation (G32E) in an evolutionary conservative domain of sPPARD remarkably increases external ear size in pigs. Here, we investigated the underlying molecular mechanism of the causal mutation at the cellular level. Using a luciferase reporter system, we showed that the G32E substitution reduced transcription activity of sPPARD in a ligand-dependent manner. By comparison of the subcellular localization of wild-type and mutated sPPARD in both PK-15 cells and pinna cartilage-derived primary chondrocytes, we found that the G32E substitution promoted CRM-1 mediated nuclear exportation of sPPARD. With the surface plasmon resonance technology, we further revealed that the G32E substitution had negligible effect on its ligand binding affinity. Finally, we used co-immunoprecipitation and luciferase reporter assays to show that the G32E substitution greatly reduced ubiquitination level by blocking ubiquitination of the crucial A/B domain and consequently decreased transcription activity of sPPARD. Taken together, our findings strongly support that G32E is a functional variant that plays a key role in biological activity of sPPARD, which advances our understanding of the underlying mechanism of sPPARD G32E for ear size in pigs.

## Introduction

Deciphering the genetic architecture of complex traits is a big challenge for geneticists. To date, only a handful of causal variants underlying quantitative traits have been unequivocally identified in domestic animals [1], such as the *DGAT1* K232A [2]–[4] and *ABCG2* Y581S [5] mutations affecting milk in cattle, the porcine *IGF2* intron3 g.3072 G>A [6] and the ovine *MSTN* 3′UTR g.6723 G>A [7] mutations influencing muscle mass, and *PLAG1* variants affecting bovine stature [8]. Recently, we have successfully identified a missense mutation in the peroxisome proliferator-activated receptor beta/delta (PPARD) that contributes to external ear size in pigs [9]. The protein-altering mutation explains a proportion of ear size across diverse Chinese pigs. It has been well characterized that PPARD plays a pivotal role in lipid metabolisms, embryonic development, inflammatory response, wound healing and cancer in human and mice [10]–[12]. Our findings further suggest a novel biological role of PPARD in external ear development. We also show that the causative substitution reduces the expression of β-catenin and its target genes, which is likely favorable for enlarged ear size [9]. However, the detailed cellular mechanism of the substitution remains largely unknown and warrants further investigations. Here, we systematically tested the effect of the G32E substitution on transcription activity, subcellular localization, ligand binding affinity and post-translational activity of porcine PPARD (sPPARD). Our results clearly show that G32E is a functional variant affecting activity of sPPARD, providing novel insights into the biology of the important nuclear hormone receptor.

## Materials and Methods

### Ethics statement

All the procedures involving animals are in compliance with the care and use guidelines of experimental animals established by the Ministry of Agriculture of China. The ethics committee of Jiangxi Agricultural University specifically approved this study.

### Cell culture, vector constructs and reagents

One-generation primary chondrocytes were isolated from external ears of 1-week old piglet with the wild-type *PPARD* allele as described previously with minor modifications [13]. The one-generation chondrocytes maintained in DMEM-F12 (Gibco) were used for the subsequent experiments. PK-15 (Xiangf Bio) and HEK293T (SIBS) cells were cultured in DMEM (high glucose; Gibco). Both mediums were supplemented with 10% fetal bovine serum (Hyclone) and antibiotic solution. When cells were incubated with the ligand GW0742 (Tocris), culture mediums were replaced with DMEM plus 5% charcoal-stripped serum (Dextrax). Using the wild-type *sPPARD* full-length cDNA clone (Genechem) as template, site-directed mutagenesis generated single-amino-acid substitutions including G32E, K16R, K17R, K18R and K16-18R of PPARD by overlap extension PCR. Primers for amplification of the above products are given in Table S1. An initial denaturation step at 94°C for 1 minute was followed by 30 cycles of denaturation at 94°C for 30 seconds, annealing at 55°C for 30 seconds and extension at 72°C for 90 seconds and a final extension step at 72°C for 30 minutes. Wild-type sPPARD and G32E mutant were cloned into pEGFP-C1 expression vectors (Addgene), and wild-type sPPARD, G32E, K16R, K17R, K18R and K16-18R mutants were inserted into pcDNA4A-His expression vectors (Addgene). Reporter plasmid PPREx3-tk-Luc was generated by inserting artificially synthesized PPREx3-tk fragment into pGL4.20 reporter vector (Promega). The sequence and orientation of the generated plasmids were verified by Sanger DNA sequencing. HA tagged ubiquitin expression vector (HA-ub) was a gift of Shiming Zhao (Fudan University, China), and β-galactosidase (pCX-nLacZ) vector was kindly provided by Herui Wang (Fudan University, China). The ligand GW0742 and proteasome inhibitor PS341 (Tocris) were dissolved in Me_2_SO. Leptomycin B (Beyotime) was prepared in ethanol with a final concentration of 50 nM.

### Chondrocyte proliferation

The one-generation primary chondrocytes were seeded onto 6-well tissue culture dishes (Nunc) at 3×10^4^ cells per well in mediums as described below. Twenty-four hours later, cell numbers were measured with a counting chamber to determine plating efficiency. The remaining cells were treated in triplicate with 0.1% DMSO, 0.1 µM and 1 µM GW0742 (a specific ligand of PPARD). Cell numbers were determined at daily intervals, and the remaining cells were retreated with fresh mediums each day for up to 7 days.

### Luciferase reporter assay

PK-15 cells (1×10^5^ cells/well) were seeded in 24-well plates. When cells were 90% confluent, cells were co-transfected with pcDNA4A-His-sPPARD and –G32E mutant expression vectors, PPREx3-tk-Luc and pCX-nLacZ constructs using Lipofectamine 2000 (Invitrogen). Twenty-four hours after transfection, cells were treated with GW0742 overnight and then measured for firefly luciferase on a LUMAT LB 9507 luminometer (Berthold Technologies) and for β-galactosidase control activity on an iMark microplate absorbance reader (BioRad) according to standard protocols. The relative luciferase activity was determined by the ratio of firefly luciferase to β-galactosidase activity. Each reporter plasmid was transfected in triplicate, and the reporter assay was performed three times independently.

### Immunofluorescence staining

To characterize subcellular localization of wild-type sPPARD and mutants, one-generation primary chondrocytes were transiently transfected with pEGFP-C1-sPPARD and -G32E mutant expression vectors using Gene Pulser Electroporation Buffer (BioRad) as described previously [14]. The pEGFP-C1-sPPARD and -G32E vectors were also transiently transfected into PK-15 cells by Lipofectamine 2000 (Invitrogen) following the manufacturer's instruction. After 24 hours, cells were fixed and stained with DAPI according to a standard protocol [15]. Fluorescence images were viewed and assessed with a LSM510 Meta confocal microscope and a Zen microscope software (Carl Zeiss).

### Proteins preparation and microarray experiment

When HEK293T cells were 90% confluent, pcDNA4A-His-sPPARD and -G32E mutant expression vectors were transfected into cells in 10-cm dishes by Lipofectamine 2000 (Invitrogen). Thirty-two hours after transfection, fusion proteins were purified from cell lysate by affinity column chromatography over His-Bind resin (GE Healthcare) according to the supplier's instruction. Protein concentration was measured by the standard Bradford method [16]. The binding affinity of GW0742 towards wild-type sPPARD or G32E mutant was assessed with the surface plasmon resonance (SPR) technology [17] via a Biacore 3000 instrument (GE Healthcare) as described previously [18]. Briefly, proteins covalently bound to CM5 sensor chips were diluted into 10 mM sodium acetate buffer (pH 4.2) with a final concentration of 0.02 mg/mL, and were then immobilized to the chips using a standard amine-coupling procedure. Prior to the start of experimentation, baseline was equilibrated with a continuous flow of running buffer [10 mmol/L HEPES, 150 mmol/L NaCl, 3 mmol/L EDTA and 0.005% (v/v) surfactant P20, pH 7.4]. Five different concentrations of GW0742 were then injected into the channels at a flow rate of 30 µL/min for 1 min, followed by disassociation for 2 min. For kinetic analysis, wild-type sPPARD and G32E mutant of five reference-subtracted sensorgram signals were globally fitted into a 1∶1 interaction model [19], giving an association rate constant (*K_a_*) and a dissociation rate constant (*K_d_*). The equilibrium dissociation constant (*K_D_*) was calculated by the ratio of rate constant (*K_d_/K_a_*).

### Ubiquitination test and Western blot analysis

Expression vectors pcDNA4A-His-sPPARD, -G32E, -K16R, -K17R, -K18R or -K16-18R and HA-ub were co-transfected into HEK293T cells by Lipofectamine 2000 (Invitrogen). At 24 hour post-transfection, cells were treated with 10 µM PS341 for 4 hours or 5 µM GW0742 overnight. Then cells were lysed in a urea buffer consisting of 8 M urea, 0.1 M Na_2_HPO_4_/NaH_2_PO_4_ (pH 8.0), 0.05% Tween 20 and 10 mM imidazole [pH 8.0]. A total of 5 mg protein was incubated with His-select nickel affinity gels (Sigma) overnight. Beads were washed with urea buffer containing additional 10 mM imidazole (pH 8.0) twice and then with native wash buffer (0.1 M Na_2_HPO_4_/NaH_2_PO_4_ (pH 8.0), 0.05% Tween 20 and 20 mM imidazole (pH 8.0)). Protein was dissolved in Laemmli buffer (BioRad), loaded on 10% SDS-PAGE gels and analyzed by Western blotting with antibodies against tubulin (Beyotime), His tag (Beyotime) and HA tag (Beyotime) [20].

### Statistical analysis

Statistical analysis was performed using a two-tailed student's test. *P*-value≤0.05 was treated as significance.

## Results

### Activation of PPARD inhibits the growth *in vitro* of chondrocytes from pig external ear

PPARD is now widely accepted to suppress cartilage growth of bones. Its activation by endogenous ligands promotes chondrocyte apoptosis in growth plate [21], [22]. To determine whether activation of sPPARD affects cartilage growth of external ear, we treated one-generation pinna cartilage-derived primary chondrocytes with PPARD specific ligand (GW0742) and quantified cell numbers with a counting chamber over the 7-days culture period. The result showed that activation of PPARD by GW0742 inhibited the growth of primary ear chondrocytes of wild-type pigs *in vitro*, especially after 5-days treatment (**Figure S1**).

### The G32E substitution reduces transcription activity of sPPARD in a ligand-dependent manner

**Figure 1:**
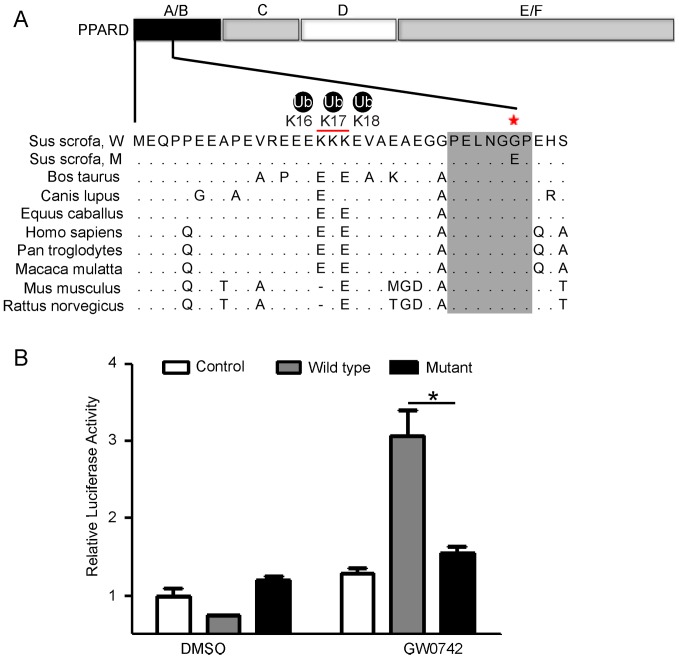
The G32E substitution prevents ligand-induced transcription activity of sPPARD. (A) Glycine 32 in the A/B domain of sPPARD is conserved across mammals as described in [9]. The asterisk and red line indicates the positions of glycine 32 and lysines 16–18. (B) PK-15 cells were transfected with pcDNA4A-His, wild-type sPPARD, and G32E mutant expression vectors along with PPREx3-tk-Luc and pCX-nLacZ reporter vectors. Cells were incubated overnight with DMSO or GW0742 (5 µmol/L). The ratio of firefly luciferase to β-galactosidase activity was defined as relative luciferase activity. Data represent -fold increase (mean ±S.D. of triplicate experiments) relative to empty vector-transfected and ligand untreated cells. * *P*<0.05 indicates significant difference between wild-type and G32E mutant sPPARD in the presence of ligand.

The transcription activity of PPARD is induced by specific ligands whose binding results in conformational change and thereby switches it towards activation state [23]. Glycine at position 32 in the A/B domain of sPPARD is a conserved amino acid across mammals, and glutamic acid exists only in Chinese large-eared pigs (**Figure 1A**) [9]. To determine whether glycine 32 is essential to the transcription activity of sPPARD, PK-15 cells co-expressing pcDNA4A-His-sPPARD or –G32E mutant and PPAR-responsive reporter (PPREx3-tk-Luc) were incubated overnight with or without the specific ligand of PPARD (GW0742). Ligand treatment significantly (*P*<0.05) increased transcription activity of wild-type sPPARD compared to the mutant, indicating that the glutamic acid 32 substitution attenuated ligand-induced transcription activity of sPPARD (**Figure 1B**). As above-indicated, reduction of PPARD activity could promote external ear growth due to losing the inhibition of sPPARD on cartilage growth.

### The G32E substitution promotes CRM1-mediated nuclear export of sPPARD

**Figure 2:**
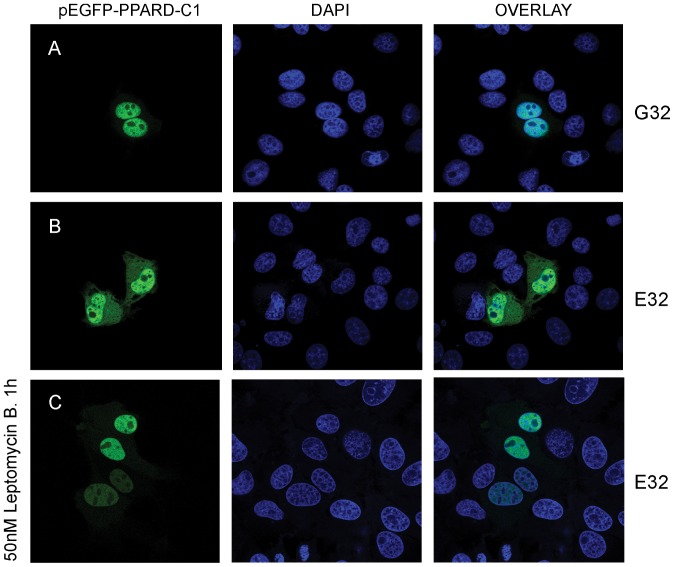
The G32E substitution stimulates CRM1-mediated nuclear export of sPPARD. PK-15 cells were transfected with pEGFP-C1-wild-type sPPARD (A), and -G32E mutant (B). G32E mutant was treated by leptomycin B (50 nM, 1 h) that blocked CRM1-dependent nuclear export (C). sPPARD subcellular localization was analyzed by GFP fluorescence at 24 hour post-transfection. Cell nuclei were counterstained with DAPI.

Subcellular compartmentalization is an important regulatory mechanism of protein activity especially for transcription factors [15]. To check whether the G32E substitution alters subcellular distribution of sPPARD, we introduced a mutation at codon 32 that changes glycine into glutamic acid in pEGFP-C1-sPPARD. After PK-15 cells were transiently transfected with wild-type or mutant constructs, wild-type receptors localized in nucleus whereas G32E mutants partly located in cytoplasm (**Figure 2A and 2B**). We obtained the same result in one-generation pinna cartilage-derived primary chondrocytes (**Figure S2**). These observations indicate that the G32E substitution promotes nuclear export of sPPARD.

von Knethen et al [15] reported that the phosphorylation of serine in the A/B domain activates nucleus export of hPPARG. However, no potential phosphorylation site was found in the A/B domain of sPPARD according to the prediction method described in [24]. We thus speculated that glutamic acid 32 at the A/B domain promotes nuclear export of sPPARD in another way. Given that chromosome region maintenance 1 (CRM1) is involved in most proteins exporting from nucleus to cytoplasm [25], we evaluated CRM1 effect on subcellular localization of G32E mutants by leptomycin B treatment that blocks CRM1-dependent shuttling [26]. The pEGFP-C1-sPPARD G32E mutant constructs were transiently transfected into PK-15 cells. After 24 hours, cells were treated 1 hour with 50 nM leptomycin B. The treatment blocked cytosolic localization of G32E mutants (**Figure 2C**). Therefore, the G32E substitution activates CRM1-mediated shuttling of sPPARD from nucleus to cytoplasm. It is known that the leptomycin B inhibits CRM1-mediated nuclear export by blocking CRM1 binding with nuclear export signal (NES) of cargo proteins [27]. We identified a classical short leucine-rich NES within C-terminal domain between amino acids 343–352 using the NetNES software (http://www.cbs.dtu.dk) (**Figure S3**). Taken together, we proposed that the G32E substitution might promote the interaction of NES of sPPARD with CRM1 to stimulate nuclear export of sPPARD.

### The G32E substitution causes negligible change on ligand binding affinity

**Figure 3:**
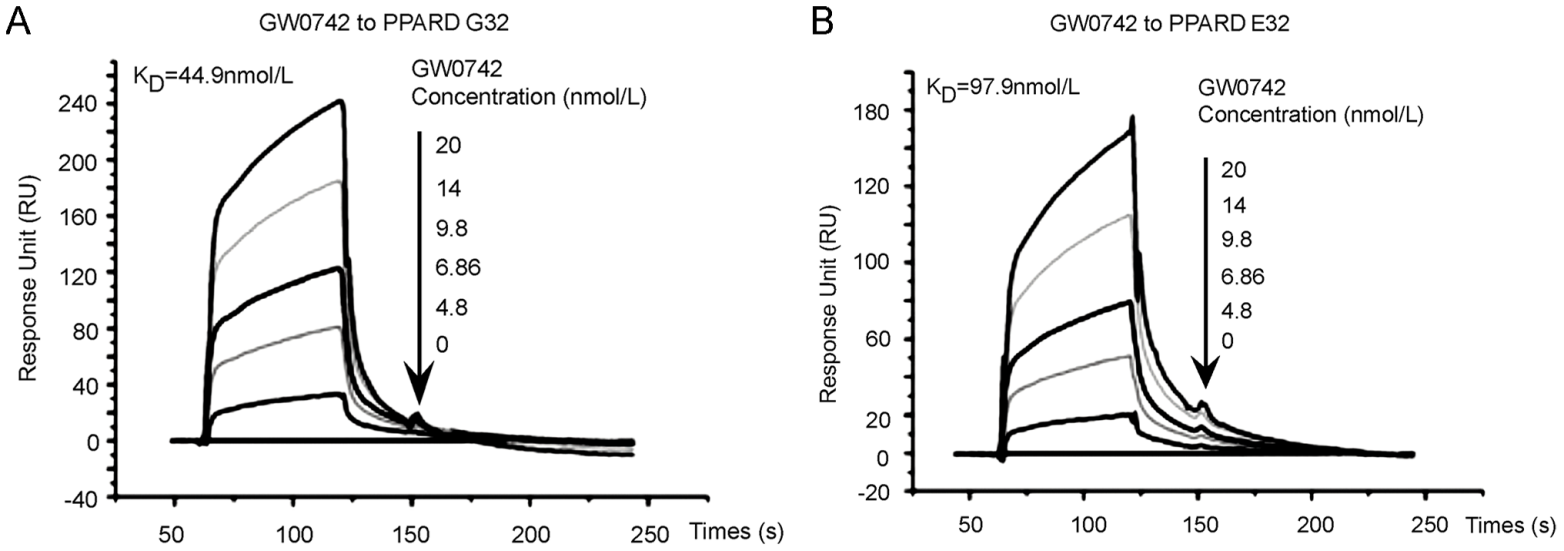
The G32E substitution does not alter the binding affinity of sPPARD with ligand. The surface plasmon resonance analysis was performed, and the sensorgrams were obtained from injection of GW0742 at 5 concentrations over surface-immobilized wild-type sPPARD (G32, A) or G32E mutant (E32, B). GW0742 were injected for 60 s, and dissociation was monitored for more than 120 s. The concentrations (µmol/L) are shown next to the arrows. For the kinetic analysis, wild-type sPPARD and G32E mutants of five reference-subtracted sensorgram signals were globally fitted into a 1∶1 interaction model [19], giving an association rate constant (*K_a_*), and a dissociation rate constant (*K_d_*). The equilibrium dissociation constant (*K_D_*) was calculated by the ratio of rate constant (*K_d_/K_a_*).

To detect whether the G32E substitution alters ligand-binding affinity of sPPARD, we performed the kinetic analysis for GW0742 binding affinity to His-tagged wild-type and G32E mutant sPPARD *in vitro* using the SPR technology. The results showed that GW0742 interacted with sPPARD in a dose-dependent manner (**Figure 3**). The binding of GW0742 to the immobilized sPPARD had the equilibrium dissociation rate (constant *K_D_*) of 44.9 nM for the wild-type sPPARD and 97.9 nM for the mutant, respectively (**Figure 3**). There was only 2-fold difference between two forms of sPPARD, thereby indicating that the G32E substitution had little effect on its ligand-binding affinity.

### The G32E substitution alters ubiquitination of sPPARD

**Figure 4:**
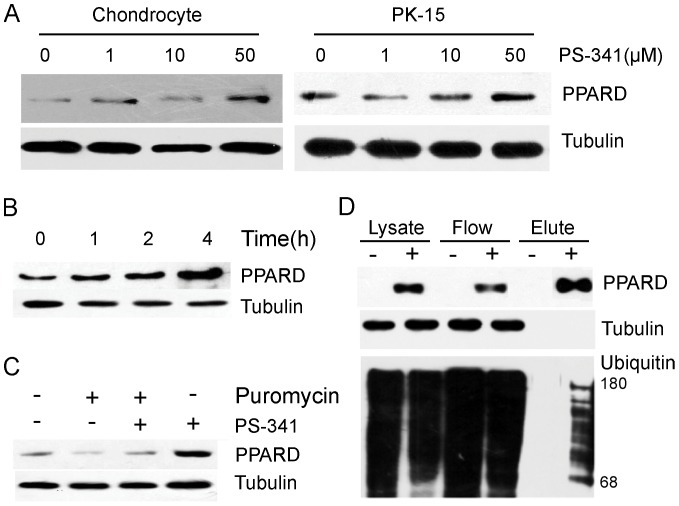
sPPARD turnover is controlled by ubiquitin-proteasome system. (A) Chondrocyte and PK-15 cells were treated with PS341 at 4 different concentrations for 4 h, and sPPARD levels were detected by Western blot. (B) PK-15 cells were treated with 10 µM PS341 and sPPARD levels were detected by Western blot at four time points after the treatment. (C) PK-15 cells were treated with puromycin, puromycin and PS341, or PS341 alone for 4 hours and sPPARD levels were detected by Western blot. (D) HEK293T cells were transfected with HA-ub vector along with pcDNA4A-His or –sPPARD expression vectors and after 24 hours were incubated with 10 µM PS341 for 4 hours. His-tagged sPPARD was pulled down with nickel affinity gel under denaturing conditions. Expression levels of sPPARD and tubulin in cell lysates are shown below. An arrow and a bar indicate mono-ubiquitinated and poly-ubiquitinated sPPARD, respectively. sPPARD was detected in cell lysate, flow-through, and eluate fractions.

Like most transcription factors, the concentration of PPARs is rigidly controlled, at least in part, through ubiquitin-proteasome pathway [28]–[30]. Human PPARD is ubiquitinated and thereby rapidly degraded by 26S proteasome, whereas ligands deubiquitinate and stabilize the receptor [28]. To determine whether sPPARD turnover is controlled by the proteasome, PK-15 cells were treated with proteasome inhibitor PS341 at 4 different concentrations for 4 h or at 10 µM for 4 different time intervals. sPPARD level was evaluated by Western blot after the treatment. The treatment appeared to cause an increased accumulation of the receptor (**Figure 4A and 4B**). Further, PK-15 cells were treated with protein synthesis inhibitors puromycin, PS341, or both compounds together (**Figure 4C**). Treatment with puromycin decreased sPPARD levels, whereas protein levels were relatively higher (*P*<0.05) in the presence of puromycin and PS341. Because the proteasome only interact with target proteins covalently attached to ubiquitin (ub) chains [31], we determined whether sPPARD was ubiquitinated. HEK293T cells were co-transfected with HA-ub and pcDNA4A-His-sPPARD expression vectors and then incubated with PS-341 for 4 hours. His-tagged sPPARD was isolated from cell lysates using nickel affinity gels. The poly-ubiquitinated sPPARD species characterized by high molecular weight were detected with HA antibody (**Figure 4D**). Altogether, we proposed that sPPARD turnover was also controlled by ubiquitin-proteasome system.

**Figure 5:**
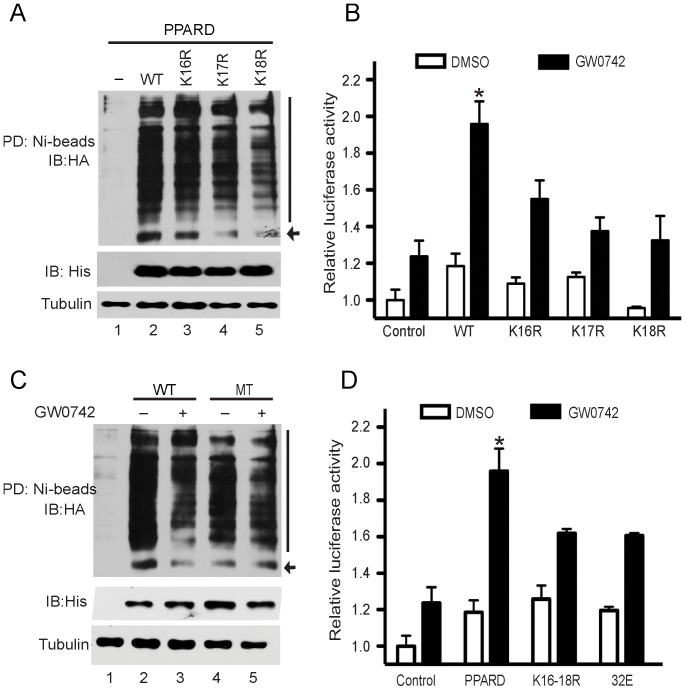
The G32E substitution attenuates ubiquitination of sPPARD and consequently reduces transcription activity of sPPARD. (A) Lysines 16, 17 and 18 are ubiquitination sites of sPPARD. HEK293T cells were transfected with pcDNA4A-His, -wild-type sPPARD (WT), -K16R, -K17R, or -K18R expression vectors along with HA-ub vector, and then after 24 hours cells were incubated with 10 µM PS341 for 4 h. His-tagged sPPARD was pulled down with nickel affinity gel under denaturing conditions. Expression levels of sPPARD and tubulin in cell lysates are shown below. An arrow and a bar indicate mono-ubiquitinated and poly-ubiquitinated sPPARD, respectively. (B) The ubiquitination in the A/B domain of sPPARD promotes its transcription activity. Luciferase activity of wild-type sPPARD (WT) and K16R, K17R, K18R mutants were assessed as described in the legend for Figure 1B. An asterisk indicates significant (*P*<0.05) difference between several forms of sPPARD. (C) The G32E substitution reduces ubiquitination of sPPARD. HEK293T cells expressing wild-type sPPARD or G32E mutant were incubated with (+) or without (-) GW0742 overnight and analyzed as mentioned above. (D) Both G32E and K16-18R mutants reduce transcription activity of sPPARD. Luciferase activity of His-tagged sPPARD, -K16-18R and -G32E mutants (32E) were assessed as described in the legend for Figure 1B. An asterisk indicates significant (*P*<0.05) difference between several forms of sPPARD.

The ubiquitin-proteasome system (UPS) has various non-proteolytic functions, such as regulating transcription activities [32]. In general, critical regulatory elements of ubiquitination are lysine residues that can covalently attach ubiquitin and thereby launch degradation procedure [33]. A bioinformatics software CKSAAP_UBSITE [34] predicted that three lysine residues at positions 16, 17 and 18 in the A/B domain were potential ubiquitination sites of sPPARD. To examine which lysine residue is an ubiquitination site, we mutated lysines 16, 17 and 18 of pcDNA4A-His-PPARD into arginine, respectively. Each vector was transfected into HEK293T cells along with HA-ub expression vector, and then after 24 hours cells were treated with PS341 for 4 hours. The ubiquitination levels of K16R, K17R and K18R mutants reduced remarkably (**Figure 5A**), showing that these lysine residues were all ubiquitination sites. To understand the effect of these sites on transcription activity of sPPARD, PPAR-responsive reporter activity were tested in PK-15 cells overexpressing wild-type or mutatant receptors after 12 hours of incubation with GW0742. Three sPPARD mutants had greatly (*P*<0.05) reduced transcription activity compared to the wild-type receptor, indicating the ubiquitination of the A/B domain is involved in gene activation (**Figure 5B**).

To determine whether the G32E substitution attenuate ubiquitination of sPPARD, we cotransfected HEK293T cells with HA-ub and two forms of His-tagged PPARD constructs (pcDNA4A-His-sPPARD and -G32E mutant constructs), which were then cultured in the absence (DMSO) or presence (GW0742) of ligand for 12 hours. The G32E mutant showed reduced ubiquitination upon both treatments compared to the wild-type receptor (**Figure 5C**). Considering that the G32E substitution is adjacent to the three above-mentioned ubiquitination sites, we compared the PPAR-responsive reporter activity of G32E mutant and K16-18R mutant in PK-15 cells (**Figure 5D**). Both receptors showed similar transcription activity, indicating that the G32E substitution might attenuate ubiquitination of lysine residue at positions 16, 17 and 18 of sPPARD.

## Discussion

PPARD is a member of the PPAR family, which includes three isoforms, namely PPAR alpha, beta/delta and gamma. They all bind with PPAR response elements (PPREs) in promoters of target genes and share a highly similar mechanism of action [35]. PPARs are composed of a N-terminal domain (A/B), a DNA binding domain (C), a hinge domain (D) and a ligand-binding domain (E/F) (**Figure 1A**) [36]. It has been documented that the constitutive phosphorylation of the A/B domain profoundly affects transcription activity of PPARs by altering their subcellular localization [15], [37]–[40], and stability via UPS-mediated degradation [40], [41]. However, no potential phosphorylation site was found in the A/B domain of sPPARD according to the prediction method described in [34]. To test if the G32E substitution in the A/B domain performs the similar function as the constitutive phosphorylation sites of serine in this domain of PPARs, we herein test the effect of the G32E substitution on the following molecular characteristics of sPPARD: subcellular localization, binding affinity to ligand and ubiquitination level.

First, we confirm that the mutation reduces ligand-dependent transcription activation of sPPARD. Second, our analysis of subcellular localization shows that the G32E substitution activates CRM1-mediated nuclear export of sPPARD. Further, we reveal a novel function of ubiquitinations in the A/B domain of sPPARD as a non-proteolytic signal, which is required for efficient ligand-induced transactivation. The G32E substitution proximal to these ubiquitins sites reduces ubiquitination of sPPARD and consequently inhibits PPARD-dependent transactivation. Our data reveals, for the first time, some ubiquitin sites of PPARs in addition to UPS influencing transcription activity, which was poorly understood before this study.

The above-mentioned findings together with our previous report [9] provide an instructive basis for the underlying mechanism of PPARD G32E affecting pig ear size. Here, we show that the G32E substitution stimulates nuclear export of sPPARD in a CRM1-mediated manner. Moreover, the substitution attenuates ubiquitination of lysine residues in the critical A/B domain of sPPARD. The two-sided effects collectively reduce the biological activity of sPPARD. In the nucleus, PPARD increases β-catenin binding to TCF/LEF transcription factors that activate transcription of target genes important for diverse cellular function including cartilage development and fat metabolism [42]. It is thereby expectable that the G32E substitution would decrease the activity of β-catenin and its target genes. The expectation is in good agreement with our previous finding that PPARD G32E mediates down-regulation of β-catenin and its downstream gene expression in pig ear-derived primary fibroblast cells [9]. WNT/β-catenin signaling has been firmly demonstrated to suppress adipogenesis [43], [44]. Altogether, we suppose that the G32E substitution reduces the activity of sPPARD and subsequently decreases the expression of β-catenin and its target genes, ultimately leading to lipid production and storage and cartilage growth that contribute to enlarged ear size in pigs. In the near future, it is warranted to further test the effect of substitution on the regulation of its target genes at a genome level using chromatin immunoprecipitation and high-throughout sequencing assays, such as ChIp-Seq and RNA-Seq. The findings would provide deeper insights into the cellular mechanism of PPARD G32E contributing to external ear size in pigs, which would ultimately benefit studies of congenital pinna defects in humans.

The genomic region harboring sPPARD G32E is of great interest in pig genetics, because significant QTL for diverse traits related to growth, carcass length, skeletal morphology and fat deposition have been consistently evidenced in this region using different crosses between Chinese indigenous pigs and Western commercial breeds [45]–[50]. Given that sPPARD serves as a crucial and multifaceted determinant of diverse biological functions including lipid metabolism, cartilage development, chondrocyte proliferation and differentiation [51]–[54], sPPARD is a strong candidate of the multiple significant pleiotropic QTL on SSC7. The molecular characterization of PPARD G32E might also contribute to dissect potential pleiotropic effects of sPPARD on growth, carcass and fatness traits in pigs.

## Supporting Information

Table S1 **PCR primer sequences.**
(DOC)

Figure S1 **Ligand activation of sPPARD leads to inhibition of chondrocytes growth.** Pinna cartilage-derived primary chondrocytes were seeded onto 6-well tissue culture dishes and cultured over 7 days in the presence of 0.1% DMSO, 0.1 µM and 1 µM GW0742. Cell numbers were quantified daily with a counter chamber. Values represent the mean ±S.E.M. from triplicate and independent samples. An asterisk (*) indicates significant (*P*<0.05) difference between the GW0742-treated group and the DMSO-treated control.
(TIF)

Figure S2 **The G32E substitution in the A/B domain of sPPARD activates cytosolic localization in primary chondrocyte cells.** Cells were transfected with pEGFP-C1-wild-type sPPARD (A) and -G32E mutant (B) by electroporation. sPPARD subcellular localization was analyzed by GFP fluorescence at 24 hour post-transfection. Cell nuclei were counterstained with DAPI.
(TIF)

Figure S3 **A classical short leucine-rich nuclear export signal was identified between amino acids 343–352 in the C-terminal E/F domain of sPPARD by the NetNES software.** NES score was calculated from the Markov Model (HMM) and Artificial Neural Network (NN) scores. If the NES score exceeds the threshold, those amino acid residues are predicted to be a nuclear export signal.
(TIF)
