# Supplementary material for: The G32E Functional Variant Reduces Activity of PPARD by Nuclear Export and Post-Translational Modification in Pigs
Source: PLoS One. 2013 Sep 18;8(9):e75925. doi: 10.1371/journal.pone.0075925 (PMC3776753; doi:10.1371/journal.pone.0075925)
Supplement: Table S1 — PCR primer sequences. (DOC) [file pone.0075925.s001.doc]

Table S1

| Name of primers | Forward primer (5’-3’) | Reverse primer (5’-3’) |
| --- | --- | --- |
| G32E | GTGCTCTGG**TT**CCCCATTGAGCTCTGGGCCTCC^1^ | TCAATGGGG**AA**CCAGAGCACTCGCTTCCCTCC |
| K16R | AGA**G**GAAAAAGGAAGTGGCAGAGGCCGA | CTTCCTTTTTC**C**TCTCCTCTTCCCGGACCTCAGG |
| K17R | AAGA**G**AAAGGAAGTGGCAGAGGCCGAAGG | ACTTCCTTT**C**TCTTCTCCTCTTCCCGGACCTCAGG |
| K18R | GAAAA**G**GGAAGTGGCAGAGGCCGAAGGAGGC | GCCACTTCC**C**TTTTCTTCTCCTCTTCCCGGACCTCAGG |
| K16-18R | AGA**G**GA**G**AA**G**GGAAGTGGCAGAGGCCGA | CTTCC**C**TT**C**TC**C**TCTCCTCTTCCCGGACCTCAGG |
| pEGFP-C1- sPPARβ | ATT*TCCGGA*ATGGAGCAGCCGCCGGAG^2^ | TTT*GTCGAC*TCAGTACATGTCCTTGTAG |
| pcDNA4A-His-sPPARβ | TTTT*GGTACCT*ATGGAGCAGCCGCCGGAGG | TTTT*TCTAGA*TCAGTACATGTCCTTGTAGATC |
| pEGFP-C1- sPPARβ-Δ1-74 | ATT*TCCGGA*CGGGTGTGCGGGGACAAGGC | TTTT*TCTAGA*GATCTCCTGGAGCAGGGGGTGCAGC |

^1^Bold indicates changed nucleoties; ^2^Italics indicates restriction site.
