# Supplementary figures and images for: The G32E Functional Variant Reduces Activity of PPARD by Nuclear Export and Post-Translational Modification in Pigs
Source: PLoS One. 2013 Sep 18;8(9):e75925. doi: 10.1371/journal.pone.0075925 (PMC3776753; doi:10.1371/journal.pone.0075925)

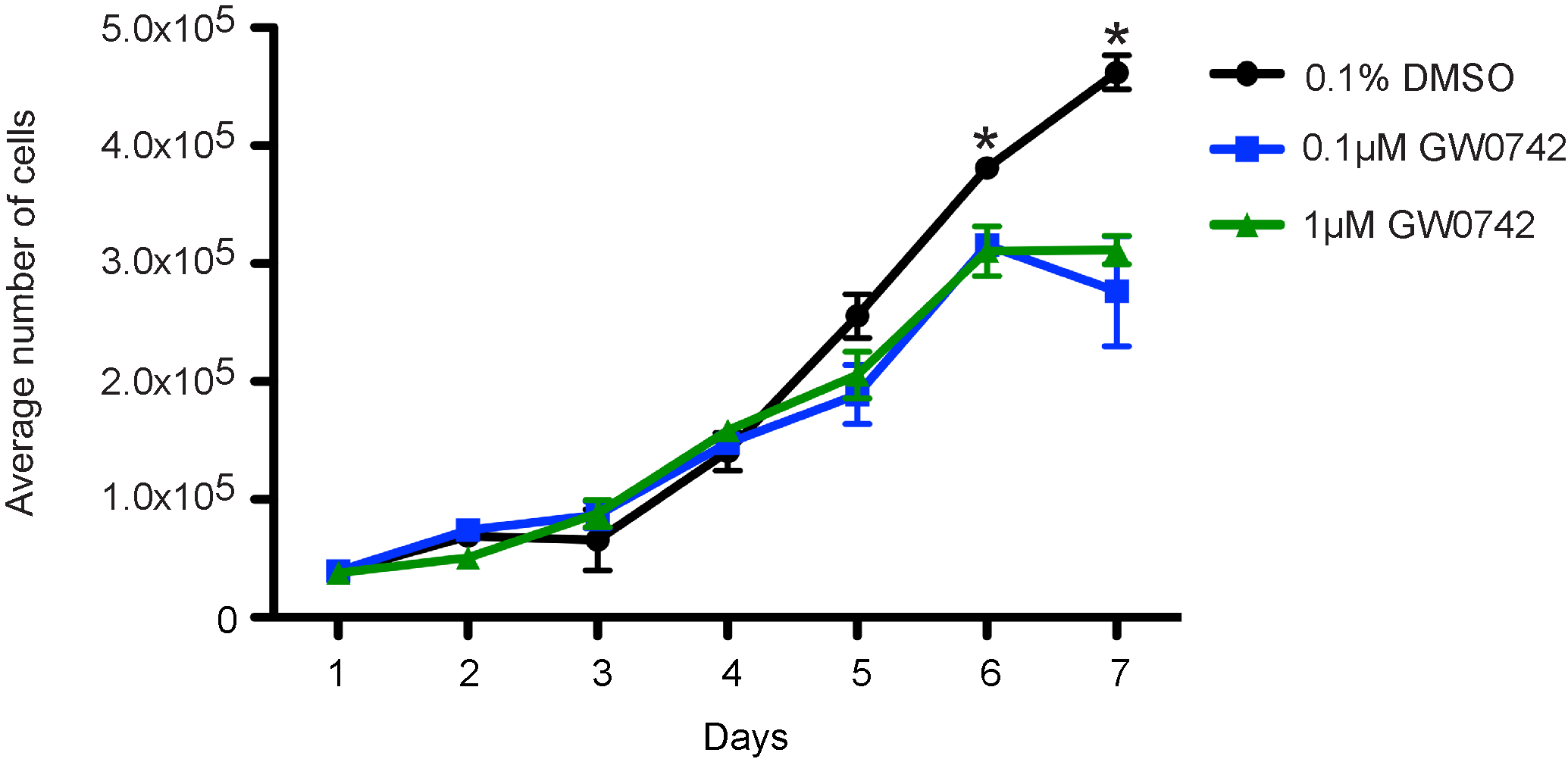

Supplement: Figure S1 — Ligand activation of sPPARD leads to inhibition of chondrocytes growth. Pinna cartilage-derived primary chondrocytes were seeded onto 6-well tissue culture dishes and cultured over 7 days in the presence of 0.1% DMSO, 0.1 µM and 1 µM GW0742. Cell numbers were quantified daily with a counter chamber. Values represent the mean ±S.E.M. from triplicate and independent samples. An asterisk (*) indicates significant (P<0.05) difference between the GW0742-treated group and the DMSO-treated control. (TIF) [file pone.0075925.s002.tif]

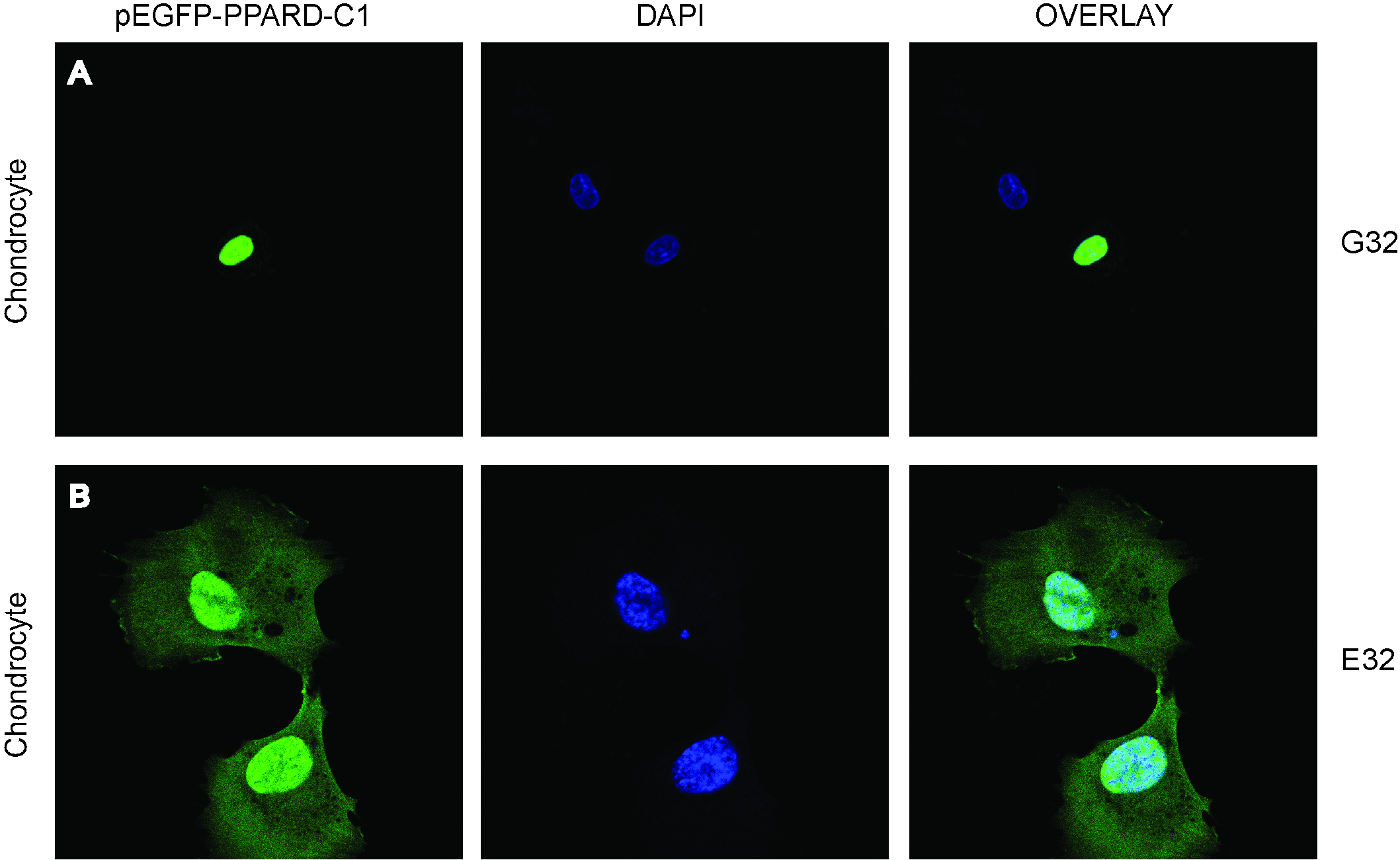

Supplement: Figure S2 — The G32E substitution in the A/B domain of sPPARD activates cytosolic localization in primary chondrocyte cells. Cells were transfected with pEGFP-C1-wild-type sPPARD (A) and -G32E mutant (B) by electroporation. sPPARD subcellular localization was analyzed by GFP fluorescence at 24 hour post-transfection. Cell nuclei were counterstained with DAPI. (TIF) [file pone.0075925.s003.tif]

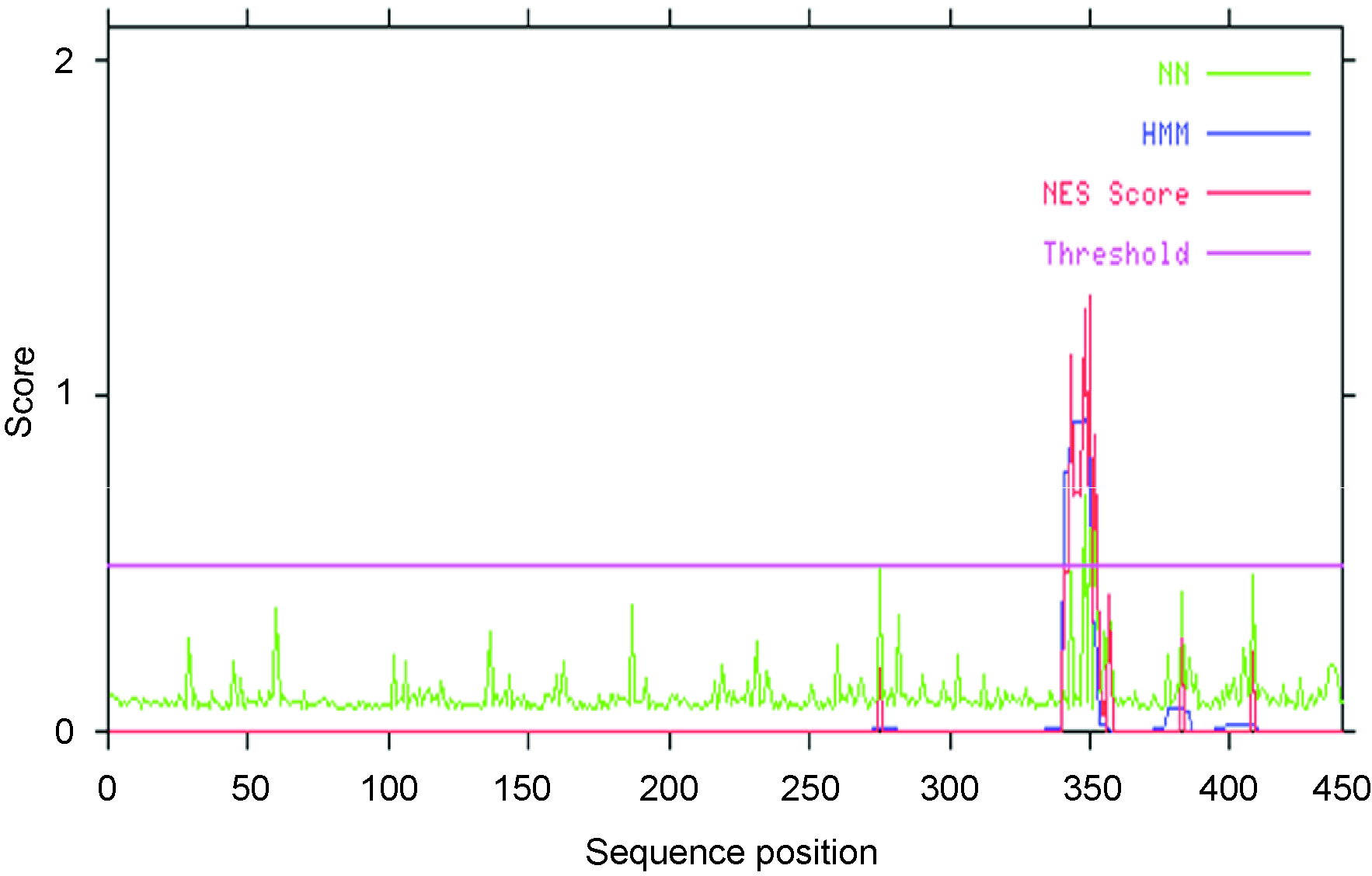

Supplement: Figure S3 — A classical short leucine-rich nuclear export signal was identified between amino acids 343–352 in the C-terminal E/F domain of sPPARD by the NetNES software. NES score was calculated from the Markov Model (HMM) and Artificial Neural Network (NN) scores. If the NES score exceeds the threshold, those amino acid residues are predicted to be a nuclear export signal. (TIF) [file pone.0075925.s004.tif]
